# Hepatitis C virus genotyping based on Core and NS5B regions in Cameroonian patients

**DOI:** 10.1186/s12985-019-1214-9

**Published:** 2019-08-09

**Authors:** Paul Alain Tagnouokam-Ngoupo, Marie Nicole Ngoufack, Sebastien Kenmoe, Simon Frédéric Lissock, Marie Amougou-Atsama, Robert Banai, Laure Ngono, Richard Njouom

**Affiliations:** Virology Department, Centre Pasteur of Cameroon, 451 rue 2005 Yaounde 2, Po Box 1274, Yaounde, Cameroon

**Keywords:** HCV, Genotyping, Core, NS5B, Genotypes, Subtypes, Cameroon

## Abstract

**Background:**

Current HCV treatments are genotype specific although potential pan-genotype treatments have recently been described. Therefore, genotyping is an essential tool for the therapeutic management of HCV infection and a variety of technologies have been developed for HCV genotypes determination. Sequences analysis of HCV sub-genomic regions is considered as gold standard and is widely used for HCV genotyping. Here, we compared HCV genotyping using core and NS5B regions in routine practice in HCV-positive Cameroonian patients.

**Methods:**

All plasma samples received at Centre Pasteur of Cameroon (CPC) in 2016 for HCV genotyping were included. Viral loads were determined using the Abbott Real Time assay. Further, genotyping was based on the amplification and sequencing of core and NS5B regions following by phylogenetic analysis of corresponding sequences.

**Results:**

A total of 369 samples were received during the study period with high viral load values (median: 930,952 IU/ml; IQR: 281,833-2,861,179). Positive amplification was obtained in at least one genomic region (core or NS5B) for all the samples with similar amplification rate in the two genomic regions (*p* = 0.34). Phylogenetic analysis showed that among the 369 samples, 146 (39.6%) were classified as genotype 4, 132 (35.8%) as genotype 1, 89 (24.1%) as genotype 2, in both core and NS5B regions. Interestingly, for two samples (0.54%) discordant genotypes were obtained in both regions with the core region classified as genotype 4 while the NS5B was identified as genotype 1 indicating the presence of putative HCV recombinant virus or multiple infections in these samples. Discrimination of HCV subtypes was most likely possible with NS5B compared to core region.

**Conclusions:**

We found high amplification rates of HCV in both core and NS5B regions, and a good concordance was obtained at genotype level using both regions except for two samples where putative 1–4 recombinants/multiple infections were detected. Therefore, HCV genotyping based on at least two genomic regions could help to identify putative recombinants and improve therapeutic management of HCV infection.

## Background

Hepatitis C virus (HCV) is classified into seven genotypes, 67 confirmed subtypes and 20 provisionally assigned subtypes [1, 2]. Genotypes 1–3 are common worldwide, while genotypes 4 and 5 are mostly found in lower-income countries. Genotypes 1, 2 and 4 have been shown to co-circulate in Cameroon [3, 4]. There is no vaccine against HCV, but many HCV treatment options and cure rates have greatly increased in the last decade with the development of direct-acting antivirals (DAA) [5]. Most often these DAA therapies are genotype specific although some pan-genotype HCV treatments have recently been described and approved by stringent regulatory authorities [6–8]. Actually, these pan-genotypic drugs are mostly used in developing countries and in clinical trials because of their high costs [9]. Despite the opportunities to access low-price generic medicines are increasing, treatment of HCV infection in developing countries relies on genotype specific DAAs (“https://www.who.int/hepatitis/news-events/hep-c-access-report-2018-key-messages/en/”). Therefore, genotyping is an essential tool for the therapeutic management of HCV infection and a variety of technologies have been developed for HCV genotypes determination.

Restriction fragment length polymorphism (RFLP) analysis has been developed for some HCV genomic regions: the 5′ non-coding and the contiguous core regions. The choice of these genomic sequences was based on their relative nucleotide homology among different genotypes and the presence of polymorphic sites [10]. RFLP is performed using endonucleases that will break down the DNA amplicons at specific sites, different HCV genotypes and subtypes will therefore be illustrated by RFLP digestion patterns on agarose gel. However, a mutation occurring in one of the polymorphic sites recognized by the restriction endonuclease could be could be limiting for HCV genotyping and subtyping [10].

Other methods are based on the 5′ untranslated (5′-UTR) region because of high conservation within genotypes but considerable variability between subtypes [11]. One of these methods is the line probe assay (LiPA), which is a reverse-hybridization assay based on variations found in 5′-UTR regions of the different hepatitis HCV genotypes and subtypes. The commercial test Versant HCV genotype assay developed by Siemens Laboratories is one of the most widely used LiPA methods for HCV genotyping. However, although LiPA is considered to be a good method for HCV genotyping, it is less suitable for HCV subtype’s determination [12].

Today, amplification of HCV sub-genomic regions (core, E1 and NS5 regions), followed by sequences analysis is considered as gold standard and is widely used for HCV genotyping [13, 14]. This method allows accurate identification of the subtype and is used for epidemiological studies of circulating virus strains. NS5B is the most common target region, however, some studies have shown a higher amplification rate of Core region compared to NS5B [15]. In addition, recombinant HCV viruses, with mosaic genome derived from two different genotypes have recently been described in Cameroon and abroad with discordant genotypes in both core and NS5B regions [16–20]. These intergenotypic recombinants involved genotypes 1 and 2, 1 and 4, and 2 and 5. This recombination could be problematic for therapeutic management particularly if the DAA anti-viral treatment responses vary among genotypes and therefore, point-out the limitation of HCV genotyping based only on one genomic region [18, 21, 22].

In the present study, we compared HCV genotyping using core and NS5B regions in routine practice in HCV-positive Cameroonian patients in order to highlight potential impact on their therapeutic management. HCV Viral load was performed on all samples followed by direct sequencing of core and NS5B regions. Phylogenetic analyses using generated HCV sequences of both regions allowed the identification of genotypes as well as subtypes.

## Methods

### Study population and HCV viral load

The Centre Pasteur of Cameroon (CPC) is the reference laboratory for HIV and Hepatitis in Cameroon. As part of this role**,** patients are routinely referred to CPC for viral load and genotyping. Data reported here were collected in the framework of these routine activities and no additional parameter was assessed.

From January to December 2016, all samples received at CPC for HCV genotyping and viral load were included. Demographic data were recorded for all patients. The HCV viral loads were determined using the Abbott RealTime HCV assay and the Abbott m2000 platforms (Abbott molecular, Wiesbaden-Germany) according to the manufacturer’s instructions. Briefly, 0.5 mL plasma sample (from EDTA tubes) was used for RNA extraction on the Abbott m2000sp followed by amplification using the m2000rt. The detection limit was 12 IU/mL.

### Amplification and sequencing of Core and NSB regions

For HCV genotyping, total RNA was extracted from 140 μL of plasma using QIAamp® Viral RNA Mini Kit (Qiagen, Courtaboeuf, France), according to the manufacturer’s instructions, following by amplification of Core and NSB regions using nested polymerase chain reaction (nested-RT PCR).

For Core region, a fragment (400 nt) was amplified using primer pairs previously described by Lole et al. [23] with slight modifications on Core OS, Core IS and Core IAS (Table 1) reported by Njouom et al. [24]. The mixture of 10 μL RNA and 2 μL (150 ng/μL) of random hexamer (NNNNNN) was used in the denaturation step at 70 °C for 10 min following by the generation of cDNA at 42 °C for 90 min using 8 U of AMV Reverse Transcriptase (Promega Corporation, USA) and 200 μM dNTPs. PCR step was then performed in a final volume of 50 μL containing 1.5 U of Taq polymerase (Life Technologies Corporation, USA), 0.2 μM of each primer, 1.5 mM MgCl_2,_ 200 μM dNTPs and 5 μL of cDNA. A Perkin Elmer Gene Amp PCR System 9700 was used with the following cycling conditions: 94 °C for 3 min, followed by two cycles of 95 °C for 30 s, 60 °C for 30 s and 72 °C for 30 s followed immediately by the two same cycles with an auto-decrement temperature of 1 °C until 51 °C, and then 20 cycles of 95 °C for 30 s, 50 °C for 30 s, 50 °C for 30 s and final extension at 72 °C for 7 min. Nested PCR step was further performed in the same conditions as the first round PCR.

**Table 1:** Primers used for amplification of Core and NS5B regions

Genomic regions	Primers	Sequences (5′------3′)
Core	Core OS (outer f)	ACTGCCTGATAGGGTGCTTGCGAG
	Core OAS (outer r)	ATGTACCCCATGAGGTCGGC
	Core IS (inner f)	AGGTCTCGTAGACCGTGCATCATG
	Core IAS (inner r)	CAYGTRAGGGTATCGATGAC
NS5B	SO755 (outer and inner f)	TATGAYACCCGCTGYTTTGACTC
	ASO1121 (outer r)	GCNGARTAYCTVGTCATAGCCTC
	ENO2BIS (inner r)	GCTAGTCATAGCCTCCTG

f, forward and r, reverse. The modifications from the primer sequences described by Lole et al. [23] are underlined

For NS5B region, a fragment (382 nt) was amplified by RT-PCR “touch Down” followed by semi-nested PCR using the same primers as previously reported by Laperche et al. [25] and Njouom et al. [24]. RT-PCR was performed using SuperScript™ III One-Step RT-PCR System with Platinum® Taq (Life Technologies Corporation, USA) in a final volume of 50 μL containing 0.2 μM of each primer, 2.3 mM MgSO4, 200 μM dNTPs and 10 μL of RNA extract. A Perkin Elmer Gene Amp PCR System 9700 was used with the following cycling conditions: 50 °C for 30 min, 95 °C for 2 min, followed by five cycles of 93 °C for 30 s, 60 °C for 45 s, and 72 °C for 1 min), followed by 35 cycles of 93 °C for 30 s, 60 °C for 45 s, and 72 °C for 1 min with an auto-decrement temperature of 0.3 °C for annealing at each cycle. The final extension was at 72 °C for 10 min. In the second round PCR, 2 μL of RT-PCR products were used in a final volume of 50 μL containing 1.25 U of Taq polymerase, 0.2 μM of each primer, and 1.5 mM MgCl_2_. The cycling conditions consisted of 95 °C for 5 min followed by 35 cycles of 95 °C for 30 s, 55 °C for 30 s, 72 °C for 30s and a final extension step of 72 °C for 10 min.

All nested-PCR products of both core and NS5B regions were further sequenced by Sanger method using GenomeLab DTCS-Quick Start Kit (Beckman Coulter, Paris, France) according to manufacturer’s instructions.

### Determination of HCV genotypes and subtypes

Nucleotides sequences obtained in both regions were aligned by ClustalW with reference sequences representing HCV genotypes and subtypes retrieved from Genbank (http://www.ncbi.nlm.nih.gov/genbank/). Phylogenetic trees were further constructed in MEGA 6.06 software by the neighbor-joining method with the Kimura two-parameter method for computing evolutionary distances [26]. Robustness of the tree topologies was estimated by bootstrap analysis with 1000 pseudo-replicate data sets, and only bootstrap values > 70 were considered significant.

### Statistical analysis

Statistical analysis was performed with the R version 3.4.1 software. *P*-value ≤0.05 determined with Fisher’s Exact Test or Pearson chi-square test was considered significant.

## Results

### Samples and viral load

A total of 369 samples were received during the study period with 212 (57.5%) from females. The median age of the patients was 62 years [Interquartile range (IQR): 56–69 years]. Globally, high viral load values were obtained with a median viral load of 930,952 IU/ml (IQR: 281,833-2,861,179) or 6 LogIU/ml (IQR: 5.4–6.5). No information on treatment was available for all the patients.

### Amplification rate of Core and NS5B regions

A positive amplification was obtained in at least one genomic region (core or NS5B) for all the samples with similar amplification rate in the two genomic regions (*p* = 0.34). Three hundred and forty six (93.8%) samples were positive in the core region while 338 (91.6%) were positive in the NS5B region. Three hundred and fifteen samples were positive in both regions (85.4%), 31 (8.4%) samples positive in core region only and 23 (6.2%) samples positive in the NS5B region only (Table 2**)**.

**Table 2:** Amplification rate of Core and NS5B regions

		Core region		
		Positive	Negative	Total
NS5B region	Positive	315	23	338 (91.6%)
	Negative	31	0	31 (8.4%)
	Total	346 (93.8%)	23 (6.2%)	369 (100%)

Amplification rates varied with viral load in both core and NS5B regions (Table 3), but the difference did not reach statistical significance (*p* = 0.07) indicating that there is a high probability to have a positive result for HCV genotyping in Cameroonian patient using the primers and methods described here.

**Table 3:** The amplification rate of Core and NS5B regions according to HCV viral loads

		Number of positive samples	
Viral loads (IU/mL)	Samples (n)	Core, n (%)	NS5B, n (%)
<1000	2	2 (100)	2 (100)
1000–10,000	6	6 (100)	6 (100)
10,000–100,000	33	32 (97)	31 (93.9)
100,000–1,000,000	146	126 (86.3)	132 (90.4)
1,000,000–10,000,000	176	174 (98.9)	161 (91.5)
>10,000,000	6	6 (100)	6 (100)
Total	369	346 (93.8)	338 (91.69)

IU/mL, international units per milliliter

### Performance of HCV genotyping using Core and NS5B sequence analysis

**Fig. 1:**
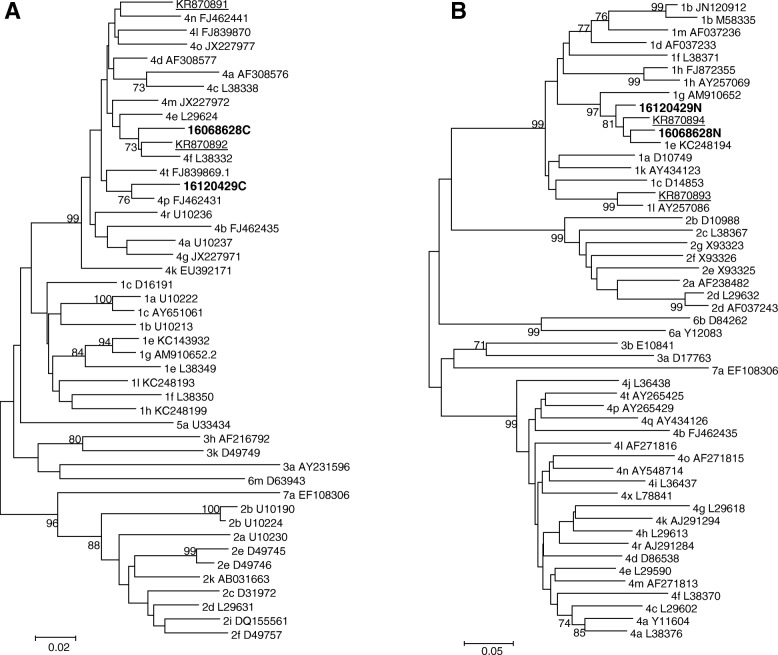
Phylogenetic trees of the sequences derived from the core and NS5B regions of the putative recombinant viruses described in this work. The neighbour-joining tree is based on the core (**a**) and NS5B (**b**) sequences. The sequences of putative recombinant viruses described here are shown in bold. Previously reported recombinants in Cameroon are underlined [18]. The reliability of the tree topologies was estimated by bootstrap analysis with 1000 pseudoreplicate data sets and for clarity, all bootstrap less than 70 have been omitted

Phylogenetic analysis showed that among the 369 samples, 129 (39.6%) were classified as genotype 4, 132 (35.8%) as genotype 1, and 89 (24.1%) as genotype 2, in both core and/or NS5B regions. Among all, genotypes were assigned in both core and NS5B for 129 samples for genotype 4, 107 samples for genotypes 1 and 77 samples for genotypes 2 (Table 4). For 10 other samples for genotype 1, 8 for genotype 2 and 5 for genotype 4, classification was based on NS5B only, while for 15 samples (genotype 1), 5 (genotype 2) and 11 genotype (4), genotyping was based on core region only (Table 4). Interestingly, for two samples (0.54%) discordant genotypes were obtained in both regions with the core region classified as genotype 4 while the NS5B was identified as genotype 1 indicating the presence of putative HCV recombinant viruses or multiple infections in these samples (Table 4). No phylogenetic relationship was found between the two sequences nor with previously described recombinant strains indicating the presence of two distinct viruses in both patients (Fig. 1). Due to the large number of sequences obtained in this study, only sequences of the two putative recombinant viruses were shown in the phylogenetic trees. However, all the sequences were submitted to the Genbank database (Accession numbers: MN208824-MN209169 for core sequences and MN208486-MN208823 for NS5B sequences).

**Table 4:** HCV genotypes classification in core and NS5B regions

	Core				
NS5B	Genotype 1	Genotype 2	Genotype 4	No amplication	Total
Genotype 1	107		**2**	10	119
Genotype 2		77		8	85
Genotype 4			129	5	134
No amplication	15	5	11		31
Total	122	82	142	23	369

Putative recombinants/multiple infections are shown in bold

Regarding the classification of HCV at the subtype level, subtype 4f was the more prevalent (22.55%) followed by subtype 1e (11.68%). Core and NS5B were not able to assign subtype to a relatively high proportion (175/369; 47.4%) and were considered as not classified (nc) namely 2nc (23.64%), 1nc (13.32%) and 4nc (10.6%). Among the 346 sequences obtained in the core regions, subtype 1e was identified in 49 (14.2%), 4f in 11 (3.2%), 1b in 2 (0.58%), 1 h in 1 (0.29%). For 281 (80.2%) sequences, the subtype was not determined (nc) among which, 4nc in 129 (45.9%), 2nc in 82 (23.7%) and 1nc in 70 (20.23%) samples. In the NS5B regions, more subtypes were identified and 80 (23.67%) were classified as 4f, 22 as 1 l (6.51%), 13 as 4p (3.85%), 10 (2.96%) as 1 h, 8 (2.37%) as 4 t, 2 (0.59%) as 2a, 2 as 1b (0.59%) and finally subtypes 1c, 4c and 4o were identified in 1 (0.30%) sample each. NS5B was not able to assign subtype for 198 samples (58.6%) among which 1nc (84; 42.4%), 2nc (83; 41.9%) and 4 nc (31; 15.7%).

Among the 281 samples not classified by core region, 1 sample was classified as subtype 1c, 9 as 1 h, 22 as 1 l, 2 as 2a, 70 as 4f, 1 as 4o, 13 as 4p and 7 as 4 t by NS5B region (Table 5). Conversely, among the 198 samples not classified by NS5B region, 38 were identified as subtype 1e, 1 as 1 h and 3 as 4f by the core region.

**Table 5:** Comparison of genotyping with core and NS5B sequences

		Core									
NS5B	1b	1e	1 h	1nc	2nc	4c	4f	4p	4nc	No amplication	Total
1b	2										2
1c				1							1
1 h				9						1	10
1 l				22							22
1nc		38	1	34				**1**	**1**	9	84
2a					2						2
2nc					75					8	83
4c						1					1
4f							7		70	3	80
4nc							3		27	1	31
4o									1		1
4p									13		13
4 t									7	1	8
No amplication		11		4	5	1	1		9		31
Total	2	49	1	70	82	2	11	1	128	23	369

nc, not classified. Samples with discordant genotypes in core and NS5B regions (putative recombinants or multiple infections) are shown in bold and those were the subtype was not determined in one region but classified by the second region are underlined

## Discussion

Current DAA for HCV treatment are genotype specific and treatment duration can vary among genotypes. Therefore, HCV genotyping is essential for the therapeutic management of HCV infection and a variety of technologies have been developed for HCV genotypes determination. However sequences analysis of HCV sub-genomic regions (core, E1 and NS5 regions) is considered as gold standard and is widely used for HCV genotyping [10].

In this study, amplification rates were high and similar for core and NS5B regions (93.8 and 91.6% respectively). Other studies have reported different results with a higher amplification rate for the core region compared to NS5B [15]. The core region is highly conserved and produces fewer false negative results in PCR reactions for HCV detection. Difficulty with NS5B region could sometimes be due to inadequate primer design, choice of highly polymorphic annealing sequences as well as low viral load [13].

Regarding amplification rate based on HCV viral load, we found no difference in both core and NS5B regions. In China, Cai et al. reported that amplification rate of both core and NS5B regions was correlated with viral load with a significant amplification rate of the core region when the viral load is ≥1.E+ 03 IU/ml [15]. This situation could be explained by different HCV genetic diversity in both countries where genotypes 1, 3 and 6 are present in China, while genotypes 1, 2 and 4 are found in Cameroon.

HCV has a high genetic variability and our result is consistent with the genetic diversity of the virus in Cameroon where genotypes 1, 2 and 4 are found [3, 4]. The predominance of genotype 4 followed by genotypes 1 and 2, is representative of the molecular epidemiology of HCV in Central Africa including Cameroon, where genotype 4 represents more than 80% followed by genotypes 1 (12%) and 2 (4%) [1]. The great genetic diversity of HCV virus in Cameroon provides a good environment for the co-circulation of multiples genotypes and subtypes which could lead to dual infections and the emergence recombinants. We found discordant genotypes with respect to core (genotype 4) and NS5B (genotype 1) in two samples (0.54%) indicating the presence of putative HCV recombinants. However, data presented here is insufficient to establish the two sequences as recombinants because only Sanger sequencing on nested RT-PCR fragments has been performed. Therefore, multiple infections (co-infections/superinfections) is also a possibility in the two samples. Recombination can occur with HCV as reported for other RNA viruses especially HIV [27]. However, characterization of near full length genomes will be more informative and also will help to confirm the mosaic pattern of these two viruses by Simplot analysis. Two other cases involving the same genotypes were previously reported in Cameroon [15]. This could probably be explained by the fact that genotypes 1 and 4 are prevalent in Cameroon compared to genotype 2 [1, 24]. Recombination is a common phenomenon that occurs during replication of RNA viruses and this impact diagnosis, treatment and follow-up of infected patients [21]. Although, this phenomenon is rare in HCV virus, we should pay more attention on it because it is relevant for clinical management of the infection regarding prognosis, natural history, and treatment recommendations as well as viral response [21, 22]. Therefore, at least two regions should be used for HCV genotyping to identify recombinant viruses or multiple infections and help to improve the therapeutic management of infected patients.

In this study, genotypes 1 and 4 were more heterogeneous with several subtypes: 1b, 1e, 1 h, 4c and 4f for core region and 1b, 1c, 1 h, 4c, 4f, 4o, 4p and 4 t for NS5B (Table 5). Discrimination of HCV subtypes was most likely possible with NS5B compared to core region because more samples (80.2%) were not classified at subtype level by core compared to 58.6% with NS5B. Similar findings were reported for 5’non coding region, where identification of HCV genotypes was possible while classification at subtype level was not possible in this region [25]. This could be due to the fact that the NS5B gene has the highest phylogenetic signal compared to all the other HCV genes [3]. Therefore NS5B region is more suitable in epidemiological studies compared to core region [3]. Murphy et al. in their study also suggest NS5B genotyping as an effective tool for studying the molecular epidemiology of HCV [28]. No intersubtype recombinant was identified, however, for some few samples, the subtypes was determined in one region and not classified in the other region.

Our study has some limitations because no information on the treatment was available for all participants. The high proportion of not classified samples at subtype level for both core and NS5B regions could be due to the lack of reference sequences of some subtypes in the phylogenetic analysis. Analyzing these samples by another comparator such as LiPA would help to establish whether the method presented here is the best suited for genotyping/subtyping in Cameroonian patients. Regarding the two samples with discordant genotypes in the core and NS5B regions, multiple infections (co-infections/superinfections) is also a possibility because only Sanger sequencing on nested RT-PCR fragments has been performed. Data presented here is insufficient to establish the two sequences as recombinants.

## Conclusion

In this study, we found high amplification rates of HCV in both core and NS5B regions, and a good concordance was obtained at genotype level using both regions except for two samples where putative 1–4 recombinant strains were detected. Since treatment option and duration are genotype specific HCV genotyping based on at least two genomic regions could help to identify putative recombinants/multiple infections and improve therapeutic management of HCV infection. However, even though recombination is rare in HCV infection, further studies are necessary to investigate clinical impacts of HCV recombinants.

## Data Availability

The nucleotide sequences of HCV described in this study were submitted to the Genbank database and accession are MN208824-MN209169 for core sequences and MN208486-MN208823 for NS5B sequences.

## Abbreviations

5′-UTR: 5′ untranslated
DAA: Direct-acting antivirals
HCV: Hepatitis C virus
IU/mL: International units per milliliter
LiPA: Line probe assay
Nc: not classified
NS5B: Non-structural protein 5B
RFLP: Restriction fragment length polymorphism
